# The Predictors of Awareness of Sexual Abuse and Sexual Violence in the Media and the Influence on Actions of the Individuals

**DOI:** 10.3389/fpsyt.2021.789144

**Published:** 2022-01-05

**Authors:** Engie Frentzen, Doris Reisacher, Elmar Brähler, Miriam Rassenhofer, Jörg M. Fegert, Andreas Witt

**Affiliations:** ^1^Department of Child and Adolescent Psychiatry/Psychotherapy, University of Ulm, Ulm, Germany; ^2^Department for Psychosomatic Medicine and Psychotherapy, University Medical Center of Johannes Gutenberg, University of Mainz, Mainz, Germany; ^3^Integrated Research and Treatment Center Adiposity Diseases, Behavioral Medicine Research Unit, Department of Psychosomatic Medicine and Psychotherapy, University of Leipzig Medical Center, Leipzig, Germany; ^4^Competence Center Child Abuse and Neglect, Kompetenzzentrum Kinderschutz in der Medizin Baden-Württemberg, Department of Child and Adolescent Psychiatry/Psychotherapy, University of Ulm, Ulm, Germany; ^5^Prevention Area of Mental Health in Baden-Württemberg, University of Ulm, Ulm, Germany

**Keywords:** sexual abuse, sexual violence, sexual assault, media, actions

## Abstract

**Introduction:** The number of reportings on sexual abuse (SA), sexual violence (SV) cases covered in the media has risen a significant amount with most cases involving women and children. The aim of the study is to explore the questions: Are people aware of sexual abuse and sexual violence in the media? What are the predictors of awareness of sexual abuse and sexual violence? Does the awareness of sexual abuse and sexual violence in the media affect the actions of the individuals?

**Methods:** A representative survey of the German-speaking resident population (2020) on physical and mental well-being was used. The participants (*N* = 2,503: females = 50.9%) were between the ages of 14 and 97 (*M* = 49.81). The German version of the Adverse Childhood Experiences Questionnaire, The General Habitual Well-Being Questionnaire and questions about own experiences of *sexual harassment on the internet*, experiences of *domestic sexual abuse* and different *socio-economic variables* were used. The outcome variables regarding the *awareness of SA and SV in the media*, different types of scandals (*church, pedophile, USA*), *#MeToo-debate* and the *change in actions* were used. Frequency analyses and binary regressions were conducted.

**Results:** One thousand five hundred and fifty-five (62.6%) respondents answered *yes* to being *aware of SA and SV in the media*. The results show that *females, aging, number of children in household, Protestant* and *Catholic religion, school graduation*, own experience(s) of *sexual harassment on the internet*, own experience(s) of *SA in childhood*, and *Adverse Childhood Experiences* have a significant higher association to the *awareness of SA and SV in the media*. *German nationality* and *Muslim religion* have a significant lower association. The variables that most commonly affected the *awareness of SA and SV, scandals, debate* and the individual *actions* were *age*, own experiences of *sexual harassment on the internet* and the *Protestant religion*.

**Conclusion:** Advertising more support centers, hotlines and linking this information to sexual abuse cases covered in the media should be considered. Media bystander interventions could be helpful to train people to react appropriately. Further investigation that considers the different types of media and its influence on the awareness of SA and SV is needed.

## Introduction

According to the World Health Organization and their definition of sexual violence (SV), it is stated that SV is considered as any sexual act including the attempt to obtain a sexual act from someone and unwanted sexual comments. This also includes the intention to traffic, against a person's sexuality using coercion, performed by any person regardless of their gender or relationship to the victim, taking place anywhere and in any setting; including but not limited to home and work environments (1). Sexual abuse (SA) is unwanted sexual activity that is performed by and with perpetrators using force, and by making threats or taking advantage of victims not able to give consent (2).

The number of cases of sexual violence recorded by the police in Germany increased in the last years (3). In a European study, the incidents of the perpetrators of non-partner sexual violence since the age of 15 shows that 23% of the perpetrators are not known to the victims before (4). There has been an increase in the likelihood of victims and third parties reporting sexual abuse to authorities, with the perpetrator being publicly well-known, or being a family member or friend. As a result of this, the number of reportings and sexual abuse cases covered in the media has risen a significant amount with most cases involving women and children (5). A study conducted in the US involving college students (18–29) shows that there are many factors that are associated with sexual abuse (SA) and sexual violence (SV), such as economic and ethnic factors that help maintain a culture of violence mostly against women (6). Additionally, SA and SV and the effects during the Covid-19 pandemic can also be seen in newer developments (7, 8). A different study suggests that the risk of intimate partner violence including SV is higher in individuals who come from and have lower educational and economic status (9). This may also stem from past common behaviors where women and victims alike were often blamed for the sexual violence they experienced (10). Although there are many factors (gender, ethnicity, and education) related to SA and SV, based on the results it would be important to know if these factors could also be a predictor of the awareness of SA and SV in the media. To our knowledge, there are no other studies that investigated (the predictors of) the awareness of SA and SV in the media.

The conversation surrounding SA and SV has picked up and has become less taboo in the last 10 years. A particular event that sparked attention and awareness of SA in Germany was the scandal in 2010, concerning sexual abuse at an educational institution which triggered the disclosure of many other sexual abuse cases in Germany (11). In addition to the scandal, the legal situation in Germany regarding sexual abuse and sexual violence has improved, and women have been given more rights since the late 1990s (12).

Allroggen et al. conducted a study in 2016 that aimed to explore the prevalence of sexual violence in men and women in Germany. The study found that more women (1.2%) reported experiencing SV compared to men (0.6%), in the last 12 months of the study being conducted (13). According to the analyses of the WHO, using data from over 80 countries showed that worldwide 35.6% of the women have experienced either physical and/ or sexual intimate partner violence or non-partner sexual violence (14). Data collected in the USA shows that, 43.6% of women and 24.8% of men experienced some type of SV (15). Generally, it is more likely for women to have one or more experiences with SA and SV (16). Although this study speaks about SA and SV in the media, this specific type of media: geolocation-based dating websites and applications has not been covered but may also play a major role in SA and SV against women (17). On the other hand, the younger generations are more encouraged to share their experiences which may be attributed to school prevention programs, and information sessions on sexuality (18). As stated in the findings of the previous studies presented, women are more often victims of SA and SV, compared to men. Additionally, there are more studies that focus on women victims and less about SA and SV inflicted on men. The investigation surrounding are people aware of sexual abuse and sexual violence in the media? What are the predictors of awareness of sexual abuse and sexual violence? Have helped to sustain the focus of this study which includes both male and female participants.

Attention by the media and public awareness has helped to shed light on the ongoing issue of sexual abuse and victimization altogether (19). A recent representative and longitudinal German study found that younger women between the ages of 18–29 have a significant and increased prevalence of the disclosure of SA. The same study states that these women may have been triggered by their awareness of topics concerning SA and SV, especially *via* social media and the #MeToo-debate in 2016–2018 (20). Therefore, media coverage of cases of SA and SV is important as 99% of the German population aged 14 and over are reached daily by media (21). Also the use of technology is becoming more frequent with ~3 h a day, on average (22). A study made up of a German population (*N* = 485) showed that the average use of social media specifically was 80.83 min (23). Social media is used more frequently by and has the greatest impact on the younger generation (24).

The #MeToo-debate was started and circulated on social media *via* Twitter (25). #MeToo aims to encourage women, children and men to speak about their own personal experiences, while holding sexual abusers accountable for their actions (26). The #MeToo-debate is only one of the various movements made by other activists who encourage the use of hashtags and other forms of advocacy to express outrage and hurt about sexual harassment in Hollywood and in the business world (27). In light of the #MeToo-debate, there have also been investigations on sexual harassment in the workplace, with many employees stating that sexual harassment is an issue in their workplace, also including those who have had a personal experience(s) of sexual harassment; along with the potential effects the #MeToo-debate may have on individuals and their roles in the future (28–30). The #MeToo-debate is an example of how individuals with the same or similar experiences as others are more likely to talk about their own experiences of sexual abuse or sexual violence, but also in how bystanders might be inspired to step in. The “bystander effect” is the effect that in a crucial situation or a situation that would be considered an emergency, the likelihood of people helping decreases with the number of people present during or witnessing the event (31). According to the results of a study conducted in 2011, those who knew someone who was being sexually assaulted was another point that was related to engaging in more positive bystander behaviors and attitudes (32). The coverage of cases of sexual abuse and sexual violence in the media are important to continue the general conversation, to inform others and to make known that SA and SV is an ongoing issue. In this way, the awareness of SA and SV can only increase in both women and men, especially in situations when witnessing an act of SA or SV. This can create a culture of positive bystander behavior. Therefore, whether the awareness of sexual abuse and sexual violence in the media affects the actions of the individual was investigated in this study.

A study conducted in Sweden that included adolescents (12–20 years) showed that 9.26% of the girls had experience(s) with online sexual harassment, 26.7% of the girls had offline experience(s) and 12.53% had both online and offline experience(s). Compared to the girls, boys are less often victims of sexual harassment (5.73% online, 20.7% offline, and 12.53% both online and offline) (33). A study conducted in 2018 showed that 34% of the recruited Australian participants had experiences with sexual harassment *via* social media (34). Additionally, adolescents that have been exposed to sexually explicit material on the internet (that have not yet received negative feedback on social media) is related to resisting the #MeToo movement, and is also related to their high credibility of rape myths and the idea of women as sexual objects (35). Maas et al. (36) indicated that people with riskier online sexual experience(s) compared with people of less risky online sexual experience(s) predicted real life risk and victimization later in life.

Individual awareness is not neutral nor objective. Survivors may experience distress if they see other people endorsing and normalizing abusive behaviors (37). Great components that make up individual awareness are cognitive schemata, expectations, desires, prior knowledge, and needs. The more pronounced these aspects are for the respective issue, the less inconsistent information are awarded. For example, people with an aggressive behavior are more aware of aggressive behavior in other people compared to individuals with less aggressive behavior. In addition, perception is influenced by one's own emotional state (38).

The studies that were previously mentioned and their findings are important for this research, as they all suggest that experiences with sexual abuse or sexual violence in some way, shape or form may affect the victim's actions, which might play an important role also in activating bystanders or motivate victims in their disclosure. To the best of the author's knowledge, there are no representative studies that investigate the awareness of sexual abuse and violence cases in the media. Due to this factor and because of the rise of public debates surrounding sexual abuse and sexual violence, the aim of the current study is to understand the following three main questions: **Are people aware of sexual abuse and sexual violence in the media? What are the predictors of awareness of sexual abuse and sexual violence? Does the awareness of sexual abuse and sexual violence in the media affect the action of the individual?** Additionally, the discussion on the subject is broad and may include adult sexual violence, and adult sexual violence inflicted onto children and adolescents. The three previous questions include scandals in the media and explore the attitudes and behavior of the population not directly involved.

## Methods

### Questions

**Question 1:** Are people aware of sexual abuse and sexual violence in the media?

**Question 2:** What are the predictors of awareness of sexual abuse and sexual violence?

**Question 3:** Does the awareness of sexual abuse and sexual violence in the media affect the action of the individual?

### Data Collection

The survey was conducted by USUMA, a leading social research institute in Germany. The questionnaire was made for a representative survey of the German-speaking resident population on physical and mental well-being that was collected in 2020. The goal was to recruit 2,500 face-to- face interviews. Therefore, through random sampling 5,676 were selected to participate. Two rounds of recruitment were carried out. In the first round, 2,015 participants were recruited and in the second round, 488 participants were recruited. In total, 2,503 participants (females = 50.9%, males = 49.1%) were included in the study. All participants were randomly selected and at first, they were verbally informed about the study and were provided with background information of the research. Experienced and trained interviewers were used for the survey. For underage participants (14 and over), at least one parent was to consent on their behalf. Written informed consent was collected and the participants were aware of their right to resign at any given time. Those who did not provide informed consent were not included in this study. Ethical approval for the study was obtained from the Ethics Committee of the Faculty of Medicine University Leipzig.

### Measures

Different scales were used for the overall questionnaire and some questions were self-generated. For this research study, the socio-economic factors, the German version of the Adverse Childhood Experiences Questionnaire (ACE-D) (39), The General Habitual Well-Being Questionnaire (FAHW-12) (40) and own experience(s) of sexual harassment on the internet were included. Additionally, the questions regarding sexual abuse and violence in the media, different scandals (pedophile, Church, USA), the #MeToo-debate and the questions regarding actions are important and relevant in order to answer the research questions.

#### Socio-Economic Variables

The following socio-economic variables were included in this study: gender, age, number of children living in your household, nationality, religion and school education.

In the questionnaire, gender was assessed using the following question “gender of the target person/subject.” The option to answer was binary, separated into two categories- male and female.

Age was assessed by using the question “When were you born? Year of birth?”. Additionally, the age in full years was calculated in a second step based on the information participants provided.

The survey was made within a German-speaking resident population, so most of these individuals filling out the questionnaire are German with a German nationality. Therefore, the variable was grouped into German and Other Nationalities. The option “other nationality” could be specified. The options that the participants had in order to answer this question were “German” and “Other, which:” with the possibility to fill out their own nationality.

Religion was determined by the answers that were given to the question “which religion do you belong to?” The categories that were provided were Protestant, Catholic, Muslim, Other and No Religion.

Level of education was assessed using the question “which school graduation have you obtained?”

There were 8 options listed for different types of academic levels including the option for participants to write a different academic level that was not listed. The German school system is diverse; therefore, many different options were given. The options were then categorized to make four groups. No graduation stayed as its own group and was made up of those that did not obtain any kind of graduation certificate, Basic and Middle Graduation was the second group that included those that obtained any of the following certificates: a secondary school leaving certificate, 10th grade polytechnic high school leaving certificate, and technical school leaving certificate. University Entrance Qualification/University Graduation was made up of those that obtained a general or subject-linked university entrance qualification, completed university, and college or university of applied sciences studies certificate and the last group was still in school.

#### German Version of the Adverse Childhood Experiences (ACE-D)

A history of adverse childhood experiences was assessed using the German version of the Adverse Childhood Experience (ACE-D) instrument (39). These experiences are divided into emotional abuse, physical abuse, sexual abuse, emotional neglect, physical neglect, separation from a parent, violence toward the mother, substance abuse by a household member, mental illness of a household member and imprisonment of a household member. The answer options in each question are dichotomous (yes or no). For the German version of the ACE-D questionnaire, a satisfactory reliability coefficient Cronbach's α = 0.76 is shown. The item separation coefficients were in a low to medium range (0.30 and 0.65), which is related to the construction of the ACE-D questionnaire since each item captures a range of stressful experiences. Thus, there are items that are relatively independent of other items (Phi = 0.10–0.58), such as mental illness of a household member, separation from a parent, and also sexual abuse (41). For the analysis, a sum score was made. Therefore, the answer “no” received the number 0 and the answer “yes” received the number 1. In general, it was possible to reach a sum score starting at 0–10.

Furthermore, a special focus was placed on the item regarding sexual abuse. In this regard, the questionnaire included the following question: Has an adult or person at least 5 years older ever touched or fondled you in a sexual way or caused you to touch their body in a sexual way? Or attempted to have or actually had oral, anal, or vaginal intercourse with you? As more analyses were conducted, this item was then labeled SA in childhood.

#### General and Habitual Well-Being (FAHW-12)

The FAHW-12 is a short questionnaire on general well-being based on the original questionnaire on general and habitual well-being (FAHW) (40). The General Habitual Well-Being Questionnaire (FAHW-12) addresses well-being, which includes aspects of both well-being and indisposition concerning physical, psychological, and social aspects. The individual items of the FAHW-12 were answered on five-point Likert scales. The correlations of the individual items with the FAHW-12 sum scale ranged from 0.159 and 0.781. A total score was calculated. For this purpose, the statements on discomfort (items 3, 4, 5, 9, 10, and 12) were subtracted from the sum of the statements on well-being (items 1, 2, 6, 7, 8, and 11) (40).

#### Sexual Harassment on the Internet

The self-generated question on sexual harassment on the internet asks: have you ever been sexually harassed on the internet? The answers options were dichotomous and were to be answered with either a yes or no.

#### Questions Concerning Sexual Abuse and Sexual Violence

All the following questions are self-generated. First, participants were asked if they were aware of SA and SV in the media. If yes, they were to answer a couple of more specific follow-up questions. These follow-up questions were concerning specific cases of SA and SV that were at some point covered in the media, along with questions regarding the participant's thoughts and actions.

##### Awareness of Sexual Abuse and Sexual Violence in the Media

The self-generated question regarding the awareness of sexual abuse and sexual violence in the media asks: Are you aware of the issue of sexual violence and abuse in the media? The answer options were dichotomous and could be answered with a yes or no. This question is a filter question. All following questions are dependent on the awareness of SA and SV. To answer these questions, the participants would have had to answer “yes” to the question concerning the awareness of SA and SV.

##### Awareness of the Different Scandals/Cases/Debates of Sexual Abuse and Violence Reported in the Media

The question were you aware of SA and SV in the media which in light of the options could have been answered with a “yes” or “no,” is followed by the question if aware of SA and SV in the media, what case/scandal/debate do you remember? The options given consisted of 11 sexual abuse scandals in the media along with an option for other, where the participants could write any other sexual abuse case in the media that they have heard of that was not listed. The abuse cases options were as follows: the Staufen abuse case, the Lügde abuse case, Darknet Forum Elysium, #MeToo-debate, abuse by clergymen in the USA, abuse by clergymen in Ireland, abuse by clergymen in Australia, abuse by clergymen in Germany, Turner case Larry Nasser in the USA, the Harvey Weinstein case and the Jeffrey Epstein case.

The scandals were grouped together based on their similar themes. For instance, the church scandals in the USA, Ireland, Australia and Germany were all grouped together, because they all consisted of SA and SV in the church setting. The variable church cases is made up of these scandals. The Darknet Forum Elysium, the Staufen and Lügde abuse cases were grouped together because they dealt with pedophilia and this group makes up the variable pedophile cases. The Harvey Weinstein case, the Jeffrey Epstein case and the Turner case Larry Nasser in the USA were grouped together because all those three cases happened in the USA and dealt with sexual abuse. These scandals make up the variable USA cases. Although, it could be argued that the #MeToo-debate is related to the Harvey Weinstein case, for this research the #MeToo-debate was left alone as its own group since it is in itself, a debate and not a case. This is known as the variable #MeToo-debate.

##### The Awareness of Sexual Abuse in the Media and Its Effect on Actions

The first question concerning the actions of the individual is: Did you feel inspired to share your experiences (if any) with others? There were five response options: (1) yes, I have shared my own experience(s) on social networks, (2) Yes, I have shared my own experience(s) with one or more person(s), (3) I wanted to, but I did not, (4) No I did not share my experience(s), and (5) I have had no experience(s) with SA or SV. Furthermore, the options stated above were categorized into two groups: yes and no. The option yes includes “Yes, I have shared my experience(s) on social media” and “Yes, I have shared my own experience(s) with one or more person(s).” The option no includes “I wanted to, but I did not” and “No I did not share my experience(s).” The fifth option “I have had no experience(s) with SA or SV” was not considered for further evaluation. In the following text this variable will be referenced to as inspired to share your experience(s).

The second question also regarding the actions of the individuals is: “The media debate has inspired me to pay more attention to sexual violence against children in my environment.” There are four options to answer this question. The answers are a ranking, which included “not inspired,” “rather not inspired,” “rather inspired,” and “inspired.” The authors grouped “rather inspired” and “inspired” into one group and “not inspired” and “rather not inspired” into another group. This variable will be referenced to as inspired to pay attention to SA or SV against children.

### Statistical Analyses

The analyses were conducted using SPSS version 27. To account for possible distortion in representativeness due to the sampling procedure, weights were used for all analyses. The survey ensures that each household in which target persons live has the same probability of being sampled. Once the household is selected, individuals in larger households have a smaller probability of selection than individuals in small households. This effect must be compensated with weights. The significance level was set at *p* < 0.05. The multicollinearity between the independent variable was explored. Two different type of analyses were used. To show the multicollinearity between the metrics independent variable, the Pearson's correlation was used. For the correlation between all the nominal variables and between the metrics and nominal variables, the Cramer's Phi was used.

To answer if people are aware of sexual abuse and violence in the media, frequency analyses of the awareness of sexual abuse in the media and the awareness of the different scandals or debates were conducted.

For the second research question “What are the predictors of awareness of sexual abuse?” frequency analyses were conducted for all socio-economic variables and predictors: gender, age, children in household, German nationality, religion, school graduation, own experience(s) with sexual harassment on the internet, General Habitual Well-Being (FAHW-12), own experience(s) SA in childhood, Adverse Childhood Experiences (ACE-D) and domestic sexual abuse. In the second step, a binary regression was conducted to analyze the association between the predictors and the awareness of SA and SV in the media. Additionally, four binary regressions were conducted to analyze the association between the predictors and the awareness of the different scandals or debate.

To answer the last research question “Does the awareness of sexual abuse in the media affect the action of the individual?” frequency analyses were conducted for the two variables concerning the actions of the individuals (Inspired to share your experience(s), Inspired to pay attention to SA or SV against children). Finally, a binary regression was conducted to analyze the association between the predictors (already mentioned above) and the actions of the individuals.

## Results

### Participants

The participants in the study were between the ages of 14 and 97 (*M* = 49.81; SD = 19.28). Out of the 2,503 participants, 1,228 were men (49.1%) and 1,275 were women (50.9%). Participants were asked about their religion and if they pertained to one; and 943 (37.7%) were Protestant, 782 (31.3%) were Catholic, 73 (2.9%) were Muslim, 54 (2.1%) belonged to another religion that was not mentioned above, and 537 (21.5%) did not belong to any religion. Of the whole study population, 98 individuals (3.9%) reported having another nationality other than German and 2,405 (96.1%) reported having a German nationality. Furthermore, 46 (1.8%) reported that they did not obtain a graduation certificate. One thousand seven hundred and ninety-three (71.7%) reported having a middle and/or high school graduation certificate and 576 (23%) reported having a college or university diploma. Eighty-eight (3.5%) reported that they were still in school. In regard to the number of children under the age of 15 living in each household, 2,123 (84.8%) reported not having any children in their household, the other 15.2% have one or more (up to 5) children in their household. Further information on sampling can be found in Table 1.

**Table 1:** Socio-economic factors of the participants.

		**Total** ***N* = 2,503**	**Female** ***N* = 1,275**	**Male** ***N* = 1,228**
Age	Mean (standard deviation)	49.81 (19.28)	50.95 (19.64)	48.62 (18.83)
Children in household	Mean (standard deviation)	0.22 (0.59)	0.24 (0.60)	0.20 (0.58)
German nationality		96.1%	97.1%	95.0%
Religion	Protestant	37.7%	41.7%	37.1%
	Catholic	31.3%	34.2%	31.3%
	Muslim	2.9%	2.5%	3.7%
	Other	2.1%	2.9%	1.6%
	No religion	21.5%	18.7%	26.3%
School graduation	No graduation	1.8%	2.2%	1.4%
	Basic and Middle Graduation	71.6%	73.7%	69.5%
	University entrance qualification/ University Graduation	23.0%	20.6%	25.5%
	Still in school	3.5%	3.5%	3.6%

### Awareness of Sexual Abuse and Violence in the Media

Regarding the question “were you aware of topics regarding sexual violence and abuse in the media?” (*N* = 2,484), 1,555 individuals (62.6%) reported that they were aware and 929 (37.4%) reported that they were not aware.

For the question “which case/scandal/debate do you remember?”, the group of cases that were most remembered were the church cases (*N* = 1,556). One thousand four hundred and forty-six individuals (92.9%) reported remembering the church cases, while 110 (7.1%) reported not remembering them. The second most remembered group of cases were the USA cases (*N* = 1,553). One thousand one hundred and sixty-four individuals (75.0%) reported remembering the USA cases, while 389 (25.0%) reported not remembering them. The third most remembered were the pedophile cases (*N* = 1,552). Nine hundred and eighty-six (63.5%) reported remembering the pedophile cases, while 566 (36.5%) reported not remembering them. The least remembered was the #MeToo-debate (*N* = 1,566). Nine hundred and eighty-eight (63.1%) reported remembering the #MeToo-debate while 578 (36.9%) reported not remembering it.

#### Predictors of Awareness of Sexual Abuse and Violence in the Media

##### Frequency Analysis

Regarding the question, “Have you ever been sexually harassed on the internet?” (*N* = 2,480) 166 (6.7%) individuals reported yes and 2,314 (83.3%) reported no. For the next question “Has an adult or person 5 years or older ever touched or caressed you sexually or made you sexually touch their body?” (*N* = 2,496), 90 (3.6%) reported yes and 2,406 (96.4%) reported no. The last question is “in a relationship, has your partner ever forced you to engage in sexual acts that you did not want to perform?” (*N* = 2,493) to which 191 (7.7%) individuals answered yes and 2,301 (92.3%) answered no.

Concerning the ACE-D score, 1,407 (56.2%) reported having no adverse childhood experiences. One thousand and twelve (40.4%) crossed off more than one to nine experiences (*M* = 1.07; SD = 1.77). The FAHW-12 had a range of −21 to +24 (*M* = 12.48; SD = 8.74).

##### Binary Regression

In the binary regression for the awareness of SA and SV in the media, the Omnibus test of the model coefficient is the Chi-squared = 202.209; *p* < 0.001, Nagelkerke *R*^2^ = 0.118, Hosmer-Lemeshow-Test *p* = 0.572 and a total percentage/classification model = 66%.

In the binary regression, the results show a significantly higher degree of awareness in SA and SV in the media in females compared to males (OR = 1.409; *p* < 0.001; 95% CI = 1.170:1.696). Aging was significantly associated with the awareness of SA and SV in the media (OR = 1.009; *p* = 0.001; 95% CI = 1.004–1.015). Other nationalities in comparison to the German nationality were associated with a lower degree of being aware of SA and SV in the media (OR = 0.498; *p* = 0.006; 95% CI = 0.302:0.820). The different religions compared to no religion show that the Protestant religion (OR = 1.277; *p* = 0.044; CI = 1.007:1.619) and the Catholic religion (OR = 1.367; *p* = 0.013; 95% CI = 1.068:1.750) were associated with a higher degree of awareness of SA and SV in the media. In contrast to this, the Muslim religion was associated with a lower degree of being aware of SA and SV in the media (OR = 0.548; *p* = 0.043; 95% CI = 0.306:0.982). Individuals with a Basic and Middle Graduation (OR = 2.868; *p* = 0.004; 95% CI = 1.398:5.883) and a University Entrance Qualification/ University Graduation (OR = 5.273; *p* < 0.001; 95% CI = 2.727:12.012) were associated with a higher degree of awareness of SA and SV in the media.

Individuals that have been harassed on the internet (OR = 1.601; *p* = 0.21; CI = 1.053:2.434) and individuals that had an experience with SA in childhood (OR = 1.183; *p* = 0.009; CI = 1.279:5.401) had a significantly lower degree of the awareness of SA and SV in the media. A higher general habitual well-being (OR = 1.034; *p* < 0.001; CI = 1.022:1.047) and Adverse Childhood Experience score (OR = 1.183; *p* < 0.001; CI = 1.108:1.263) is associated with a higher degree of awareness of SA and SV in the media. The other variables are not significant (see Table 2).

**Table 2:** Binary regression of the awareness of SA and SV in the media.

		***p***	**OR**	**95% CI**	
				**Lower limit**	**Upper limit**
Gender (RF = male)	Female	**<0.001**	**1.409**	**1.170**	**1.696**
Age		**0.001**	**1.009**	**1.004**	**1.015**
Children in household		0.137	0.891	0.765	1.037
German nationality (RF = German)	Other	**0.006**	**0.498**	**0.302**	**0.820**
Religion (RF = no religion)	Protestant	**0.044**	**1.277**	**1.007**	**1.619**
	Catholic	**0.013**	**1.367**	**1.068**	**1.750**
	Muslim	**0.043**	**0.548**	**0.306**	**0.982**
	Other	0.070	1.879	0.949	3.720
School graduation (RF = no graduation)	Basic and middle graduation	**0.004**	**2.868**	**1.398**	**5.883**
	University entrance qualification/ University Graduation	**<0.001**	**5.723**	**2.727**	**12.012**
	Still in school	0.852	1.087	0.452	2.615
Sexual harassment on the internet (RF = no)	Yes	**0.028**	**1.601**	**1.053**	**2.434**
FAHW-12		**<0.001**	**1.034**	**1.022**	**1.047**
SA in childhood (RF = no)	Yes	**0.009**	**2.628**	**1.279**	**5.401**
ACE		**<0.001**	**1.183**	**1.108**	**1.263**
Domestic sexual abuse (RF = no)	Yes	0.779	1.059	0.710	1.579

*RF, Reference category; p, p-value; OR, odds ratio; CI, confidence interval; FAHW-12, Shorter questionnaire version of the General Habitual Well-Being Questionnaire; ACE, Adverse Childhood Experiences Questionnaire. The significant values are bolded*.

In the binary regression for the awareness of pedophile cases in the media, the Omnibus test of the model coefficient is the Chi-Squared = 83.792 *p* < 0.001, Nagelkerke *R*^2^ = 0.08, Hosmer-Lemeshow-Test *p* = 0.766 and the Total percentage/classification model = 65.1%.

Age was significantly associated with the awareness of pedophile cases in the media (OR = 1.009; *p* = 0.011; 95% CI = 1.002:1.014). Additionally, the Protestant religion (OR = 1.615; *p* = 0.002; 95% CI = 1.198:2.177) and the Catholic religion (OR = 2.035; *p* < 0.001; 95% CI = 1.490:2.778) were associated with a lower degree of the awareness of the pedophile cases in the media. The Muslim religion and other religions were not significantly associated. Individuals who are still in school were associated with a lower degree of the awareness of pedophile cases in the media compared to those with no graduation (OR = 0.056; *p* = 0.003; 95% CI = 0.008:0.372). Additionally, the experience of sexual harassment on the internet was associated with a lower degree of the awareness of the pedophile cases in the media (OR = 2.429; *p* = 0.001; 95% CI = 1.463:4.035). The other variables are not significant predictors (see Table 3).

**Table 3:** Binary regression for the awareness of pedophile cases and the #MeToo-debate.

		**Pedophile Cases**				**#Metoo Debate**			
		***p***	**OR**	**95% CI**		***p***	**OR**	**95% CI**	
				**Lower limit**	**Upper limit**			**Lower limit**	**Upper limit**
Gender (RF = male)	Female	0.653	0.948	0.751	1.197	0.795	1.032	0.817	1.303
Age		**0.011**	**1.009**	**1.002**	**1.016**	**<0.001**	**0.982**	**0.975**	**0.989**
Children in household		0.700	1.043	0.843	1.290	0.760	1.035	0.830	1.290
German nationality (RF = German)	Other	0.685	1.166	0.556	2.445	0.188	1.705	0.770	3.776
Religion (RF = no religion)	Protestant	**0.002**	**1.615**	**1.198**	**2.177**	0.965	1.007	0.739	1.373
	Catholic	**<0.001**	**2.035**	**1.490**	**2.778**	0.199	0.813	0.592	1.116
	Muslim	0.627	0.801	0.327	1.961	0.713	1.210	0.437	3.350
	Other	0.052	2.118	0.994	4.513	0.112	2.043	0.846	4.929
School graduation (RF = no graduation)	Basic and Middle Graduation	0.674	1.274	0.413	3.933	0.670	1.268	0.425	3.786
	University entrance qualification/ University Graduation	0.397	1.638	0.523	5.134	0.118	2.431	0.799	7.398
	Still in school	**0.003**	**0.056**	**0.008**	**0.372**	0.015	0.176	0.044	0.713
Sexual harassment on the internet (RF = no)	Yes	**0.001**	**2.429**	**1.463**	**4.035**	**0.001**	**2.492**	**1.478**	**4.202**
FAHW-12		0.238	1.009	0.994	1.025	0.797	1.002	0.987	1.018
SA in childhood (RF = no)	Yes	0.237	0.698	0.384	1.268	0.227	0.688	0.374	1.263
ACE		0.842	1.008	0.933	1.088	0.794	1.010	0.936	1.090
Domestic sexual abuse (RF = no)	Yes	0.365	1.237	0.781	1.957	0.397	1.223	0.767	1.951

*RF, Reference category; p, p-value; OR, odds ratio; CI, confidence interval; FAHW-12, Shorter questionnaire version of the General Habitual Well-Being Questionnaire; ACE, Adverse Childhood Experiences Questionnaire. The significant values are bolded*.

In the binary regression for the awareness of the #MeToo-debate in the media, the Omnibus test of the model coefficient is the Chi-Squared = 113.130 *p* < 0.001, Nagelkerke *R*^2^ = 0.106, Hosmer-Lemeshow-Test *p* = 0.09, and the total percentage/classification model = 67.3%.

Age is associated with a lower degree of the awareness of the #MeToo-debate in the media (OR = 0.982; *p* < 0.001; 95% CI = 0.975:0.989).

Furthermore, the variable of own experience(s) with sexual harassment on the internet was the only significant predictor of being aware of the #MeToo-debate in the media (OR = 2.492 *p* = 0.001; 95% CI = 1.478:4.202). The other variables are not significant predictors for the awareness of pedophile cases in the media (see Table 3).

In the binary regression for the awareness of the church cases in the media, the Omnibus test of the model coefficient is Chi-Squared = 17.261 *p* = 0.369, Nagelkerke *R*^2^ = 0.032, Hosmer-Lemeshow-Test *p* = 0.041, Total percentage/classification model = 93.4%. Because the Chi-Square is not significant and in the Hosmer-Lemeshow test the *p*-value is <0.05, the quality of this test is low.

Age was significantly associated with the awareness of the church cases in the media (OR = 1.018; *p* = 0.008; 95% CI = 1.005:1.032). There is no further variable with a significant association to the awareness of the church cases (see Table 4). In the binary regression for the awareness of the USA cases in the media, the Omnibus test of the model coefficient is Chi-Squared = 59.429 *p* < 0.001, Nagelkerke *R*^2^ = 0.062, Hosmer-Lemeshow test *p* = 0.002 and Total percentage/classification model = 76%. Because the *p*-value in the Hosmer-Lemeshow test is <0.05, the quality of this test is low.

**Table 4:** Binary regression for the awareness of church cases and USA cases.

		**Church cases**				**USA cases**			
		***p***	**OR**	**95% CI**		***p***	**OR**	**95% CI**	
				**Lower value**	**Higher value**			**Lower value**	**Higher value**
Gender (RF = male)	Female	0.577	1.133	0.731	1.756	0.144	0.825	0.637	1.068
Age		**0.008**	**1.018**	**1.005**	**1.032**	0.518	1.003	0.995	1.011
Children in household		0.959	1.010	0.687	1.486	0.067	0.812	0.650	1.015
German nationality (RF = German)	Other	0.905	1.087	0.277	4.274	0.832	0.919	0.421	2.007
Religion (RF = no religion)	Protestant	0.768	1.091	0.612	1.946	**0.011**	**1.529**	**1.101**	**2.123**
	Catholic	0.776	1.090	0.603	1.972	**0.006**	**1.609**	**1.145**	**2.263**
	Muslim	0.431	0.569	0.140	2.316	0.837	1.109	0.416	2.953
	Other	0.599	1.502	0.329	6.846	0.210	1.708	0.739	3.945
School graduation (RF = no graduation)	Basic and Middle Graduation	0.562	1.625	0.314	8.392	0.275	1.865	0.609	5.715
	University entrance qualification/ University Graduation	0.172	3.270	0.597	17.898	**0.049**	**3.145**	**1.002**	**9.873**
	Still in school	0.193	5.788	0.413	81.167	0.368	0.526	0.130	2.126
Sexual harassment on the internet (RF = no)	Yes	0.724	1.163	0.504	2.683	**0.002**	**2.561**	**1.424**	**4.604**
FAHW-12		0.072	1.026	0.998	1.055	**0.010**	**1.022**	**1.005**	**1.040**
SA in childhood (RF = no)	Yes	0.481	0.692	0.248	1.929	0.381	0.754	0.401	1.418
ACE		0.713	1.027	0.893	1.180	0.558	0.976	0.899	1.059
Domestic sexual abuse (RF = no)	Yes	0.276	1.684	0.660	4.299	0.521	1.176	0.716	1.933

*RF, Reference category; p, p-value; OR, odds ratio; CI, confidence interval; FAHW-12, Shorter questionnaire version of the General Habitual Well-Being Questionnaire; ACE, Adverse Childhood Experiences Questionnaire. The significant values are bolded*.

The Protestant religion (OR = 1.529; *p* = 0.011; CI = 1.101: 2.123) and the Catholic religion (OR = 1.609; *p* = 0.006; 95% CI = 1.145:2.263) compared to those with no religion were associated with a higher degree of the awareness of the USA cases in the media. The Muslim religion and people of other religions compared to those with no religion are not significantly associated. Individuals with a University Graduation was associated with a higher degree (OR = 3.145; *p* = 0.049; 95%; CI = 1.002:9.873) of being aware of USA cases in the media in comparison to those with no graduation. Regarding other predicting factors, individuals who have been harassed on the internet was associated with a higher degree of the awareness of USA cases in the media (OR = 2.561; *p* = 0.002; 95% CI = 1.424:4.604).

A higher well-being score was associated with a higher degree of awareness of the USA cases in the media (OR = 1.022; *p* = 0.010; CI = 1.005:1.040). All other variables were no significant predictors of the awareness of USA cases in the media (see Table 4).

##### Awareness of Sexual Abuse in the Media and the Effect on the Action of the Individual

Out of the 420 (16.8%) persons who had answered the question “did you feel inspired to share your experiences,” 130 (31.0%) individuals felt inspired to share their experiences (if any) with others while 290 (69.0%) answered no.

Out of the 1,395 (55.7%) individuals who had answered the question regarding whether the media debate inspired them to pay more attention to SV against children in their environment, 744 (53.0%) individuals answered not at all or not that much and 652 (46.7%) answered moderate or strongly.

In the binary regression for the inspiration to share own experiences, the Omnibus test of the model coefficient is Chi-Squared = 34.128; *p* = 0.005, Nagelkerke *R*^2^ = 0.120, Hosmer-Lemeshow-Test *p* = 0.158 and Total percentage/classification model = 67.4%.

Aging is associated with a lower degree of the awareness of being inspired to share own experiences (OR = 0.981; *p* = 0.010; 95% CI = 0.967:0.995). The experience(s) of domestic sexual abuse was a significant predictor of being inspired to share own experiences (OR = 2.106; *p* = 0.031; 95% CI = 1.072:4.135). The other variables are not significant predictors for the awareness of pedophile cases in the media (see Table 5).

**Table 5:** Binary regression of the actions.

		**Inspired to share your experience**				**The media debate has inspired me to pay more attention to sexual violence against children in my environment**			
		***p***	**OR**	**95% CI**		***p***	**OR**	**95% CI**	
				**Lower limit**	**Upper limit**			**Lower limit**	**Upper limit**
Gender (RF = male)	Female	0.365	0.798	0.490	1.301	**0.007**	**1.387**	**1.094**	**1.757**
Age		**0.010**	**0.981**	**0.967**	**0.995**	0.369	0.997	0.990	1.004
Children in household		0.178	0.725	0.455	1.157	**0.009**	**1.336**	**1.074**	**1.662**
German nationality (RF = German)	Other	0.575	0.672	0.168	2.693	0.561	1.250	0.589	2.656
Religion (RF = no religion)	Protestant	0.483	0.809	0.448	1.462	**<0.001**	**2.232**	**1.626**	**3.063**
	Catholic	0.153	0.631	0.336	1.186	0.063	1.357	0.983	1.873
	Muslim	0.999	0.000	0.000		0.128	2.254	0.792	6.417
	Other	0.836	1.128	0.359	3.540	0.703	1.162	0.538	2.512
School graduation (RF = no graduation)	Basic and Middle Graduation	0.383	2.793	0.278	28.103	0.457	0.577	0.135	2.458
	University entrance qualification/ University Graduation	0.331	3.154	0.312	31.900	0.861	0.878	0.204	3.772
	Still in school	0.351	0.204	0.007	5.760	0.574	1.655	0.286	9.590
Sexual harassment on the internet (RF = no)	Yes	0.253	1.480	0.756	2.900	0.362	0.815	0.525	1.265
FAHW-12		0.871	0.998	0.969	1.027	0.747	1.003	0.987	1.018
SA in childhood (RF = no)	Yes	0.654	0.837	0.384	1.825	0.109	1.647	0.895	3.031
ACE		0.331	1.065	0.938	1.208	**0.011**	**1.106**	**1.023**	**1.196**
Domestic sexual abuse (RF = no)	Yes	**0.031**	**2.106**	**1.072**	**4.135**	0.609	1.124	0.719	1.758

*RF, Reference category; p, p-value; OR, odds ratio; CI, confidence interval; FAHW-12, Shorter questionnaire version of the General Habitual Well-Being Questionnaire; ACE, Adverse Childhood Experiences Questionnaire. The significant values are bolded*.

In the binary regression for the Inspiration to pay more attention to sexual violence against children in my environment, the Omnibus test of the model coefficient is Chi-Squared = 79.491; *p* < 0.001, Nagelkerke *R*^2^ = 0.082, Hosmer-Lemeshow test *p* = 0.506 and Total percentage/classification model = 59.2%.

Females compared to males was associated with a higher degree of the inspiration to pay more attention to sexual violence against children in their environment (OR = 1.387; *p* = 0.007; 95% CI = 1.094:1.757). Additionally, a greater number of children in the household was associated with a higher degree of the inspiration to pay more attention to sexual violence against children in their environment (OR = 1.336; *p* = 0.009; 95% CI = 1.074:1.662).

Regarding religion, those within the Protestant religion was associated with a higher degree of the inspiration to pay more attention to sexual violence against children in their environment (OR = 2.232; *p* < 0.001; 95% CI = 1.626:3.063) in comparison to people with no religion.

A higher ACE-score was associated with a higher degree of the inspiration to pay more attention to sexual violence against children in my environment (OR = 1.106; *p* = 0.011; 95% CI: 1.023:1.196). All other variables are not significant (see Table 5).

## Discussion

The aim of the present study was to report the predictors of and to examine the awareness of sexual abuse and sexual violence in the media and the influence on actions of the individuals.

### Awareness of Sexual Abuse and Violence in the Media and the Predictors

Attention by the media and public awareness has helped to shed light on the ongoing issue of sexual abuse and victimization altogether (19). The results of the current study show that the majority (62.6%) of the population is aware of SA and SV in the media which support the findings of the previous study mentioned. The discussion on SA and SV is rather broad and may include both adult sexual violence and sexual violence inflicted onto children and adolescents. In the last decades, the reporting of SA cases in the media have also risen (5). Due to the rising number of cases of SA and SV, and in the media, this could help to bring more exposure to the overall topic.However, the percentage of the awareness of SA and SV in the media is only moderately high (62.6%). Reasons for the lack of awareness (37.4%) could be that awareness is influenced by one's own emotional state. Great components that make up individual awareness are cognitive schemata, expectations, desires, prior knowledge, and needs. The more pronounced these aspects are for the respective issue, the less inconsistent information are awarded (38). This indicates that those who have experienced SA and SV are more likely to be aware of the topic in the media. Likewise, the results show that people who have experienced sexual harassment on the internet (OR = 1.601) and individuals that had an experience(s) with SA in childhood (OR = 1.183) had a significantly higher degree of the awareness of SA and SV in the media. It may be useful for those aware of sexual abuse and sexual violence, along with media cases involving SA and SV to link or inform others on where to find help or support. Although not every individual has had an experience of SA and SV, the information may be useful for them to hold on to or pass on depending if they ever encounter an experience with SA or SV, or if they know of someone who has had an experience.

More specifically, it is more likely for women to have one or more experiences with SA and SV compared to men (15, 16). This relates to our results that show a significantly higher degree of awareness of SA and SV in the media in females compared to males (OR = 1.409) which could be attributed to their personal experiences with SA and SV and could be a reason for why female gender is a predictor. Additionally, a German study showed that younger women between the ages of 18–29 have a significant and increased prevalence of the disclosure of SA. These women may have been triggered by their awareness of topics concerning SA and SV, especially *via* social media and the #MeToo-debate (20). In this study, gender has no significant association to the awareness of the #MeToo-debate as well as to the awareness of all other sexual cases (USA, Pedophile, Church) included. In order to reach a higher percentage of the male population both in Germany and in other countries, media attention and exposure should cover sexual abuse and sexual violence cases inflicted on males, as much as it does females. Perhaps this will initiate a greater conversation within the male population and bring more awareness to the fact that sexual abuse and sexual violence can happen to anyone, regardless of their gender.

The results also show that age (older age) is associated with a lower degree of the awareness of the #MeToo-debate (OR = 0.982). This indicates that younger people are often more aware of the #MeToo-debate. Further discussing age, the study additionally found that aging (getting older) is associated with a higher degree of the awareness of SA and SV, Pedophile cases and Church cases in the media (OR = 1.009; 1.009; 1.018). The different age association concerning the awareness of the #MeToo-debate could be explained by the fact that the #MeToo-debate is an online movement that has primarily circulated on social media (25). Social media is used more frequently by and has the greatest impact on the younger generation (24). Additionally, the use of media, specifically the internet increases within the ages of 14–29 (21). The results show that sexual harassment on the internet is significantly associated with a higher degree of being aware of the #MeToo-debate in the media (OR = 2.492). The awareness of the #MeToo-debate and the exposure of sexual harassment on the internet may be attributed to high internet usage. In order for the younger generation to be more knowledgeable on the topic of SA and SV, age-appropriate information could be given at schools with parental consent, or information could be given to children through parents themselves. This may assist children's awareness and help them to gain more insight on how to act if they were to encounter experiences with SA or SV.

Furthermore, another interesting result from this study concerning the religion is that religion is no significant predictor for the awareness of the Church cases in the media. This may be due to the grouping of the variable. The church cases were made up of cases in four different countries (U.S., Ireland, Australia, and Germany). It must be considered that these cases contained adult sexual violence and sexual violence and abuse inflicted onto children and adolescents, and cannot be categorized under a certain age group. Considering this sample is mostly made up of a German population belonging to different religions, their awareness of the church cases in other countries may be lower than their awareness of SA and SV in church cases within Germany. This could be a reason for why religion is not a significant predictor. Another reason for this unexpected outcome could be that the questionnaire did not assess the intensity of their belief, or the degree to which these individuals practice religion if belonging to one. A study's findings suggest that regular Catholic Church attendance is greatly declining and the number of Catholics who do not or rarely attend church/religious services is greatly increasing (42). Therefore, it is unknown if participants who specified their religion, practice or frequently attend religious services.

On the other hand, the Muslim religion is significantly associated with a smaller degree of awareness of SA and SV in the media (OR = 0.548). A study conducted in Jordan that screened for violence and abuse against mostly Muslim women found that within their sample of women who reported abuse within the questionnaire, 90% did not report the abuse to anyone (43). Therefore, the smaller degree of awareness of SA and SV in the media could be due to sexual abuse being less reported in Mosques.

### The Awareness of Sexual Abuse in the Media and the Influence on the Actions of the Individual

#### Inspiration to Share Their Own Experience(s)

In the past, women and victims alike were often blamed for the rape they experienced (10). As a consequence of that, victims may be afraid to share their experience(s) due to victim blaming. Therefore, the number of individuals who do share their experience(s) is relatively low, such as in the findings of this study. One third of the individuals felt inspired to share their experiences with others. On the other hand, very important and influential people such as celebrities who have publicly spoken out about their own experience(s) with SA and SV have helped the #MeToo-debate and the awareness of SA and SV (44). Celebrities that publicly speak out about their own experiences may allow for other individuals to see them as role models. This may in turn help to break the cycle of victim blaming by inspiring others to speak out about their own experiences as well. This may help to further increase the number of people aware of SA and SV in the media.

Additionally, aging (getting older) is associated with a lower degree of being inspired to share own experiences (OR = 0.981). This suggests that younger people are often more inspired. The younger generations are more encouraged to share their experiences. A study found that younger women in Germany between the ages of 18–29 have a significant and increased prevalence of the disclosure of SA (20). This may be because school prevention programs, and information sessions on sexuality may help to increase disclosures of SA (18).

Own experience(s) of domestic sexual abuse is a significant predictor of being inspired to share own experiences (OR = 2.106) and in a similar light, a higher ACE score is associated with a higher degree of awareness of SA and SV in the media (OR = 1.034). Therefore, being inspired to share own experiences and having a greater awareness of SA and SV in the media may be attributed to the realization and understanding of sexual abuse and sexual violence that comes with age and information on the topic. Additionally, there have been newer developments that investigate SA and SV during the Covid-19 pandemic, which may also help to shed light on the topic (7, 8). The conversation surrounding SA and SV has picked up and has become less taboo (11) which may help victims to become more informed, as more information and proper definitions of SA and SV are circulated.

#### Bystander

Furthermore, being aware of SA and SV in the media has an effect on bystanders. In this study, close to 47% answered being moderate or strongly inspired to pay more attention to SV against children in their environment. The amount of people paying attention to SA and SV in their surroundings could be increased through bystander interventions. A systematic review on bystander training programs for the prevention of SV was conducted in 2017, and the findings state that trainings can be helpful to positively change behaviors and attitudes (45). Bystander interventions could also be helpful to train people to react appropriately in crucial situations, especially involving SA and SV. Help centers for victims could also consider promoting any local bystander programs available that can be offered at any time. Furthermore, the coverage of cases in the media might increase the chances of bystanders stepping in when witnessing SA and SV, which may be due to their heightened awareness on the topic (media coverage) and the severity of the act. Other possible reasons for an increase in bystanders stepping in may be due to social approval and moral motivation (46).

Females compared to males are associated with a higher degree of being inspired to pay more attention to sexual violence against children in their environment (OR = 1.387). Awareness is influenced by one's own emotional state and experience(s) (38). Additionally, women in Germany are more often victims of SA and SV (47). Women and mothers have more frequent contact and spend more time with children compared to men and fathers (48). This could also help explain the findings of this study as to why a greater number of children in the household is associated with a higher degree of the inspiration to pay more attention to sexual violence against children in their environment (OR = 1.336). However, the number of women who are mothers and the number of men who are fathers are not known in this study's population.

The results show that individuals who feel better about their overall well-being (FAHW-12: OR = 1.034) is significantly associated with a higher degree of becoming aware of SA and SV in the media. Generally, perception is influenced by one's own emotional state (38). It is a conscious decision to use media and this may be influenced by being in a good mood. For this no reference could be found so further investigation is necessary.

ACE-experience(s) is a predictor of being inspired to pay more attention to sexual violence against children in my environment. Components of individual awareness are also knowledge (38). This could be attributed to the fact that people who have experienced negative things in their childhood, and who have experienced some kind of SA or SV are inspired to pay more attention compared to people who have had no contact or experiences. This relates to why media coverage on SA and SV is important. Not only can it help other victims speak about sexual abuse, but as the results suggest, it can also inspire them to be more aware of children experiencing SA or SV around them. Learning how to react to people who share their own experiences, along with some bystander education may help cultivate a judgement-free environment for those wanting to share their experiences with SA and SV. It must be considered that not every individual is appropriately informed on topics surrounding SA and SV, therefore the risk of victim-blaming and judgment may stem from past common behaviors, where women and victims alike were often blamed for the rape or abuse they experienced (10). Additionally, the amount of support victims get may vary in different countries.

Overall, the awareness of SA and SV in the media leads to an increased awareness in potential bystanders. In addition to bystander interventions, offers of assistance to victims (support centers, and/or hotlines) should also be disseminated more frequently through the media and linked to media cases regarding SA and SV in order to additionally reduce the number of individuals experiencing re-victimization.

### Limitation

It is to be stated that there are some limitations to this study. This study is cross-sectional, whereas a longitudinal study or randomized control trials with an intervention would have been more suitable in order to explore and to show behavioral changes. The problem in cross-sectional studies is the causal order of the variable due to the one-time survey. The necessary criterion for labeling a relationship as causal lies in the time difference between cause and effect (49). Some of the independent variables may allow for causality as they relate to childhood experiences. However, this is not true for all of them. Furthermore, the results of the evaluation show that the quality of the individual binary logistic regressions are low. The resolved variance in all regression is relatively low.

Another limitation is that the media that was used or preferred by the sample in this study was not considered. In general, this study does not collect data on the type of media and the participants' consumption behavior (e.g., hours per day). This means that the media does not differentiate between print, radio, TV or social media. Various studies show that younger people in particular are consuming more and more social media and media in general online (21, 24, 50). Social media in particular could be a danger in this respect, as filter bubbles often mean that they only reflect one's own interests (51). It is important to note that social media platforms and their algorithms are set up to show users more content based on what the user likes, shares, comments or clicks on. Therefore, it would be an invalid representation of sexual abuse in this type of media. In addition, regardless of this limitation, the study through its large randomized sample size, represents the German awareness of SA and SV in the media. Additionally, there were no further investigations on what SA and SV means to the study population. This could also explain why the awareness of SA and SV was ~50%.

It should be considered that social desirability and recollection bias may have played a role in the questions evaluated. One factor is that SA and SV, and especially one's own action in relation to this, is a sensitive topic. With sensitive topics, the tendency for social desirability increases. Furthermore, due to the personal interview, there was only a small social distance, which can also cause social desirability (52). Additionally, change in behavior (assessment of attitudes) rather than actual behavior was explored.

## Conclusion

This study shows the importance of the media reporting SA and SV cases and the influence on the individual action. The exposure of SA and SV cases in the media also leads the victims to being inspired to share their experiences. This can be beneficial to other victims who have trouble sharing their own experiences and may find comfort in knowing that there are more victims of SA and SV out there. The more cases on SA and SV covered in the media, the more likely it is that the conversation surrounding these topics will increase.

Additionally, this study can help to raise more awareness on the influence of sexual abuse and sexual violence in the media by specifically advertising more support centers, and/or hotlines and linking this important information to sexual abuse cases covered in the media in order to reduce the number of individuals experiencing re-victimization. To add, media bystander interventions or programs could be helpful to train people to react appropriately in crucial situations, especially regarding SA and SV. Nevertheless, further investigation is necessary and as for future research, it would be best to take the limitations into account, and to further prioritize the exploration and analyses of behavioral changes for the most effective results. Investigation that especially considers the different types of media and its influence on the awareness of SA and SV is needed. In particular, the influence of the scandals and the debate on behavior should be explored.

## Data Availability Statement

The dataset analyzed during the current study are available from the corresponding authors on reasonable request.

## Ethics Statement

Written informed consent was collected and the participants were aware of their right to resign at any given time. For underage participants (14 and over), at least one parent was to consent on their behalf. Those who did not provide informed consent were not included in this study. Ethical approval for the study was obtained from the Ethics Committee of the Faculty of Medicine University Leipzig.

## Author Contributions

EF and DR performed the data management, conducted the statistical analyses, and wrote the first draft of the manuscript. All authors contributed to the study conception, critically reviewed, and read and approved the final manuscript.

## Funding

The questionnaire was funded by UBSKM (Unabhängiger Beauftragter für Fragen des sexuellen Kindesmissbrauchs) as part of the accompanying research project on the Sexual Abuse Help Line led by JMF and MR.

## Conflict of Interest

The authors declare that the research was conducted in the absence of any commercial or financial relationships that could be construed as a potential conflict of interest.

## Publisher's Note

All claims expressed in this article are solely those of the authors and do not necessarily represent those of their affiliated organizations, or those of the publisher, the editors and the reviewers. Any product that may be evaluated in this article, or claim that may be made by its manufacturer, is not guaranteed or endorsed by the publisher.

## Abbreviations

95%CI: 95% confidence interval
ACE: Adverse Childhood Experiences Questionnaire
ACE-D: German version of the Adverse Childhood Experience
FAHW: General Habitual Well-Being Questionnaire
FAHW-12: Short questionnaire of the General Habitual Well-Being Questionnaire
M: Mean
SA: sexual abuse
SD: Standard Deviation
SV: sexual violence
USA: United States of America.

## References

[1] Krug EG Dahlberg LL Mercy JA Zwi AB Rafael L. World Report on Violence and Health. Geneva: World Health Organization (2002).

[2] American, Psychological Association,. Sexual Abuse. Availab;le online at: https://www.apa.org/topics/sexual-assault-harassment (accessed September 23, 2021).

[3] PKS Polizeiliche Kriminalstatistik,. Grundtabelle - ohne Tatortverteilung ab 1987. Bundeskriminalamt (2021). Available online at: https://www.bka.de/SharedDocs/Downloads/DE/Publikationen/PolizeilicheKriminalstatistik/2020/Interpretation/Faelle/ZR-F-01-T01-Faelle_xls.xlsx?__blob=publicationFileandv=4 (accessed November 4, 2021).

[4] FRA – European Union Agency for Fundamental Rights. Violence Against Women: An EU-Wide Survey. Main results Vienna: European Union (2014).

[5] Taylor LR. Has Rape Reporting Increased Over Time? (2006). Available online at: https://nij.ojp.gov/topics/articles/has-rape-reporting-increased-over-time (accessed November 4, 2021).

[6] Mellins CA Walsh K Sarvet AL Wall M Gilbert L Santelli JS. Sexual assault incidents among college undergraduates: prevalence and factors associated with risk. PLoS ONE. (2017) 12:e0186471. 10.1371/journal.pone.0186471 29117226 PMC5695602

[7] Davis R. COVID-19-related depression, anxiety, and psychological stress in sexual and gender minority populations. J Res Gender Stud. (2021) 11:9–19. 10.22381/JRGS1112021134164361

[8] Crawford S. Gender-related irritability, confusion, anger, and frustration associated with COVID-19 infection and mortality. J Res Gender Stud. (2020) 10:138–47. 10.22381/JRGS10220209

[9] Gracia E López-Quílez A Marco M Lladosa S Lila M. The spatial epidemiology of intimate partner violence: do neighborhoods matter? Am J Epidemiol. (2015) 182:58–66. 10.1093/aje/kwv016 25980418

[10] Gravelin CR Biernat M Bucher CE. Blaming the victim of acquaintance rape: individual, situational, and sociocultural factors. Front Psychol. (2018) 9:2422. 10.3389/fpsyg.2018.02422 30719014 PMC6348335

[11] Rassenhofer M Spröber N Schneider T Fegert JM. Listening to victims: use of a critical incident reporting system to enable adult victims of childhood sexual abuse to participate in a political reappraisal process in Germany. Child Abuse Neglect. (2013) 37:653–63. 10.1016/j.chiabu.2013.05.007 23796600

[12] Hellmann DF Kinninger MW Kliem S. Sexual violence against women in germany: prevalence and risk markers. Int J Environ Res Public Health. (2018) 15:1613. 10.3390/ijerph15081613 30061527 PMC6121316

[13] Allroggen M Rassenhofer M Witt A Plener PL Brähler E Fegert JM. The prevalence of sexual violence: results from a population-based sample. Dtsch Arztebl Int. (2016) 113:107–13. 10.3238/arztebl.2016.0107 26940778 PMC4791564

[14] WHO LSHTM SAMRC. Global and Regional Estimates for Violence Against Women: Prevalence and Health Burden of Intimate Partner Violence and Non-partner Sexual Violence. Geneva: WHO (2013).

[15] Smith SG Zhang X Basile KC Merrick MT Wang J Kresnow M. The National Intimate Partner and Sexual Violence Survey (NISVS): 2015 Data Brief – Updated Release. Atlanta, GA: National Center for Injury Prevention and Control, Centers for Disease Control and Prevention (2018).

[16] Walsh K Danielson CK McCauley JL Saunders BE Kilpatrick DG Resnick HS. National prevalence of posttraumatic stress disorder among sexually revictimized adolescent, college, and adult household-residing women. Arch Gen Psychiatry. (2012) 69:935–42. 10.1001/archgenpsychiatry.2012.132 22566561 PMC3474859

[17] Hughes A Kicova E Kovalova E Rowland Z. Intimate digital traces: sociosexual attitudes and behaviors on geolocation-based dating apps. J Res Gender Stud. (2020) 10:52–8. 10.22381/JRGS10120204

[18] Alaggia R Collin-Vézina D Lateef R. Facilitators and barriers to child sexual abuse (CSA) disclosures: a research update (2000-2016). Trauma Viol Abuse. (2019) 20:260–83. 10.1177/1524838017697312 29333973 PMC6429637

[19] Daigle LE. Special issue: research on sexual violence in the #MeToo Era: prevention and innovative methodologies. Am J Crim Just. (2021) 46:2–5. 10.1007/s12103-020-09601-w

[20] Witt A Jud A Finkelhor D Brähler E Fegert JM. Monitoring recent trends: the prevalence of disclosure of sexual abuse in a representative sample of the German population based on indicator 16.2.3 of the UN Sustainable Development Goals (SDG). Child Abuse Neglect. (2020) 107:104575. 10.1016/j.chiabu.2020.104575 32559553

[21] Breunig C Handel M Kessler B. Ergebnisse der ARD/ZDF-Langzeitstudie. Massenkommunikation 1964-2020: Mediennutzung im Langzeitvergleich. Media Perspektive (2020). p. 410–32. Available online at: https://www.ard-werbung.de/fileadmin/user_upload/media-perspektiven/pdf/2020/070820_Breunig_Handel_Kessler.pdf (accessed August 6, 2021).

[22] DESTATIS. Zeitverwendung (ZVE) 2012/2013. (2019). Available online at: https://www.destatis.de/DE/Themen/Gesellschaft-Umwelt/Einkommen-Konsum-Lebensbedingungen/Zeitverwendung/Tabellen/aktivitaeten-alter-zve.html (accessed September 23, 2021).

[23] Brailovskaia J Schillack H Margraf J. Tell me why are you using social media (SM)! Relationship between reasons for use of SM, SM flow, daily stress, depression, anxiety, and addictive SM use – An exploratory investigation of young adults in Germany. Comput Hum Behav. (2020) 113:1–9. 10.1016/j.chb.2020.106511

[24] Jain P. Social media addiction and its manifold consequences on younger generation. Int J Transform Media Journ Mass Commun. (2016) 1:38–46.

[25] Brünker F Wischnewski M Mirbabaie M Meinert J. The Role of Social Media during Social Movements – Observations from the #metoo Debate on Twitter. In: Proceedings of the 53rd Hawaii International Conference on System Sciences (2020). p. 2356–65. Available online at: https://scholarspace.manoa.hawaii.edu/bitstream/10125/64030/0232.pdf (accessed September 20, 2021).

[26] Murphy M. Introduction to #MeToo movement. J Fem Fam Ther. (2019) 31:63–5. 10.1080/08952833.2019.1637088

[27] Battaglia EJ Edley PP Newsom AV. Forum intersectional feminisms and sexual violence in the era of me too, trump, and kavanaugh. Forum Provoc Intersect Feminisms Sex Violenc. (2019) 42:159–71. Available online at: https://www.academia.edu/41892189/Forum_Intersectional_Feminisms_and_Sexual_Violence_in_the_Era_of_Me_Too_Trump_and_Kavanaugh

[28] Lazaroiu G Rowland Z Bartosova V. Gendered power disparities, misogynist violence, and women's oppression: the #MeToo movement against workplace sexual harassment. Contemp Read Law Soc Just. (2018) 10:57–63. 10.22381/CRLSJ10220184

[29] Rumrill J Stehel V Durana P Kolencik J. Does sexual objectification entail institutional power imbalances in organizations? Contemp Read Law Soc Just. (2018) 10:100–6. 10.22381/CRLSJ102201810

[30] Weathington B. Is sexual harassment an endemic social issue? Contemp Read Law Soc Just. (2018) 10:64–70. 10.22381/CRLSJ10220185

[31] Dorsch. Lexikon der Psychologie. Bystander Effect. (2011). Available online at: https://dorsch.hogrefe.com/stichwort/bystander-effect#search=294e3617fe85e2d76255fd5ced028667andoffset=0 (accessed November 4, 2021).

[32] McMahon S Postmus JL Koenick RA. Conceptualizing the engaging bystander approach to sexual violence prevention on college campuses. J Coll Stud Dev. (2011) 52:114–30. 10.1353/csd.2011.0002

[33] Stahl S Dennhag I. Online and offline sexual harassment associations of anxiety and depression in an adolescent sample. Nordic J Psychiatry. (2020) 75:330–5. 10.1080/08039488.2020.1856924 33347341

[34] Douglass CH Wright CJC Davis AC Lim MSC. Correlates of in-person and technology-facilitated sexual harassment from an online survey among young Australians. Sex Health. (2018) 15:361–5. 10.1071/SH17208 29852924

[35] Maes C Schreurs L van Oosten JMF Vandenbosch L. #(Me)too much? The role of sexualizing online media in adolescents' resistance towards the metoo-movement and acceptance of rape myths. J Adolesc. (2019) 77:59–69. 10.1016/j.adolescence.2019.10.005 31654849

[36] Maas MK Bray BC Noll JG. Online sexual experiences predict subsequent sexual health and victimization outcomes among female adolescents: a latent class analysis. J Youth Adolesc. (2019) 48:837–49. 10.1007/s10964-019-00995-3 30778831 PMC7135936

[37] Dworkin ER Weaver TL. The impact of sociocultural contexts on mental health following sexual violence: a conceptual model. Psychol Violence. (2021) 11:476–87. 10.1037/vio,0000350 34631201 PMC8494265

[38] Borg-Lauf M. Wahrnehmung. In: Mulot R Schmitt S, editors. Fachlexikon der Sozialen Arbeit. 8th ed. Baden-Baden: Nomos (2017).

[39] Schäfer I Wingenfeld K Spitzer C. ACE-D; Deutsche Version des Adverse Childhood Experiences Questionnaire (ACE). Fragebogen, Universität Hamburg, Hamburg (2009).

[40] Wydra G. Der Fragebogen zum allgemeinen habituellen Wohlbefinden (FAHW und FAHW-12: Entwicklung und Evaluation eines mehrdimensionalen Fragebogens. 6th ed. Saarbrücken: Universität des Saarlandes (2020).

[41] Wingenfeld K Schäfer I Terfehr K Grabski H Driessen M Grabe H. Reliable, valide und ökonomische Erfassung früher Traumatisierung: Erste psychometrische Charakterisierung der deutschen Version des Adverse Childhood Experiences Questionnaire (ACE) [The reliable, valid and economic assessment of early traumatization: first psychometric characteristics of the German version of the Adverse Childhood Experiences Questionnaire (ACE)]. Psychother Psychosom Med Psychol. (2011) 61:e10–4. 10.1055/s-0030-1263161 20878600

[42] Sander W. Religion, religiosity, and happiness. Rev Relig Res. (2017) 59:251–62. 10.1007/s13644-017-0285-6

[43] Haddad LG Shotar A Younger JB Alzyoud S Bouhaidar CM. Screening for domestic violence in Jordan: validation of an Arabic version of a domestic violence against women questionnaire. Int J Womens Health. (2011) 3:79–86. 10.2147/IJWH.S17135 21445377 PMC3061851

[44] Akoja MI Anjorin AG. Social media and women's culture of silence on sexual violence: perception of babcock university's female undergraduates. Commun Cult Africa. (2020) 2:50–76. 10.21039/cca.32

[45] Mujal GN Taylor ME Fry JL Gochez-Kerr TH Weaver NL. A systematic review of bystander interventions for the prevention of sexual violence. Trauma Viol Abuse. (2019) 22:381–96. 10.1177/1524838019849587 31204606

[46] Krämer B Schindler J. Media effects on bystander intervention: the role of exemplification, framing, risk perception, and motivations. J Interpers Violence. (2021) 36:11–2. 10.1177/0886260518808848 30379109

[47] PKS Polizeiliche Kriminalstatistik,. Vergewaltigung, sexuelle Nötigung und sexuelle Übergriffe: Übersicht nach Bundesländern. Bundeskriminalamt (2019). Available online at: https://www.bka.de/DE/AktuelleInformationen/StatistikenLagebilder/PolizeilicheKriminalstatistik/PKS2019/InteraktiveKarten/04VergewaltigungSexNoetigung/04_VergewaltigungSexNoetigungBundesrespublik.pdf;jsessionid=354384F37F6B6B77385CAD744EBFF5C4.live612?__blob=publicationFile&v=2 (accessed September 27, 2021).

[48] Craig L. Does father care mean fathers share? Gender Soc. (2006) 20:259–81. 10.1177/0891243205285212

[49] Schnell R Hill PB Esser E. Methoden der empirischen Sozialforschung. 9th ed. München: Oldenburg Verlag (2011).

[50] Twenge JM Martin GN Spitzberg BH. Trends in U.S. Adolescents' media use, 1976–2016: The rise of digital media, the decline of TV, and the (near) demise of print. Psychol Popular Media Cult. (2019) 8:329–45. 10.1037/ppm0000203

[51] Kitchens B Johnson SL Gray P. Understanding echo chambers and filter bubbles: the impact of social media on diversification and partisan shifts in news consumption. MISQ. (2020) 44:1619–49. 10.25300/MISQ/2020/16371

[52] Bogner K Landrock U. Antworttendenzen in standardisierten Umfragen. Mannheim, GESIS - Leibniz Institut für Sozialwissenschaften (SDM Survey Guidelines) (2015). 10.15465/sdm-sg_016

